# Endovascular Aneurysm Repair for a Patient with Horseshoe Kidney and the Importance of Watershed Sign and Volumetry by Preoperative Contrast-Enhanced Computed Tomography

**DOI:** 10.3400/avd.cr.21-00068

**Published:** 2021-12-25

**Authors:** Kazuma Handa, Tomohiko Sakamoto, Yumi Kakizawa, Mutsunori Kitahara, Shinya Fukui, Yukitoshi Shirakawa, Hiroyuki Nishi

**Affiliations:** 1 Department of Cardiovascular Surgery, Osaka General Medical Center, Sumiyoshi, Osaka, Japan

**Keywords:** endovascular aneurysm repair, abdominal aortic aneurysm, horseshoe kidney

## Abstract

We report a case of endovascular aneurysm repair (EVAR) in a patient with horseshoe kidney (HSK) in whom preoperative contrast-enhanced (CE) computed tomography (CT) showed watershed sign. This sign enabled prediction of postoperative renal function by accurate renal volumetry. A 75-year-old man with HSK and a 59-mm abdominal aortic aneurysm was referred for treatment. Preoperative CECT showed watershed lines at the margin of the isthmus, which was perfused by the accessory renal arteries. Using this sign, we calculated the accurate volume of the isthmus, which was 24.5% of the total parenchyma. EVAR was safely performed without renal dysfunction.

## Introduction

Horseshoe kidney (HSK) is an uncommon congenital anomaly of renal development and is thought to occur in only 0.12% of all cases of abdominal aortic aneurysm (AAA).^1)^ Open AAA repair by graft replacement for patients with HSK is difficult and requires multiple strategies for dealing with the isthmus and multiple arterial and venous anatomical anomalies.^2)^ Endovascular aneurysm repair (EVAR) has become the recognized standard and safe method of treating AAA. Stroosma et al. concluded that EVAR is preferable to open surgery in patients with HSK if the anatomy is suitable.^2)^ However, covering accessory renal arteries (ARAs) while performing EVAR may cause complications, such as renal dysfunction.^3)^ We report a case of EVAR for a patient with HSK in whom preoperative contrast-enhanced (CE) computed tomography (CT) showed the interesting sign of watershed lines. This finding enabled the prediction of postoperative renal function by renal volumetry.

## Case Report

**Figure 1:**
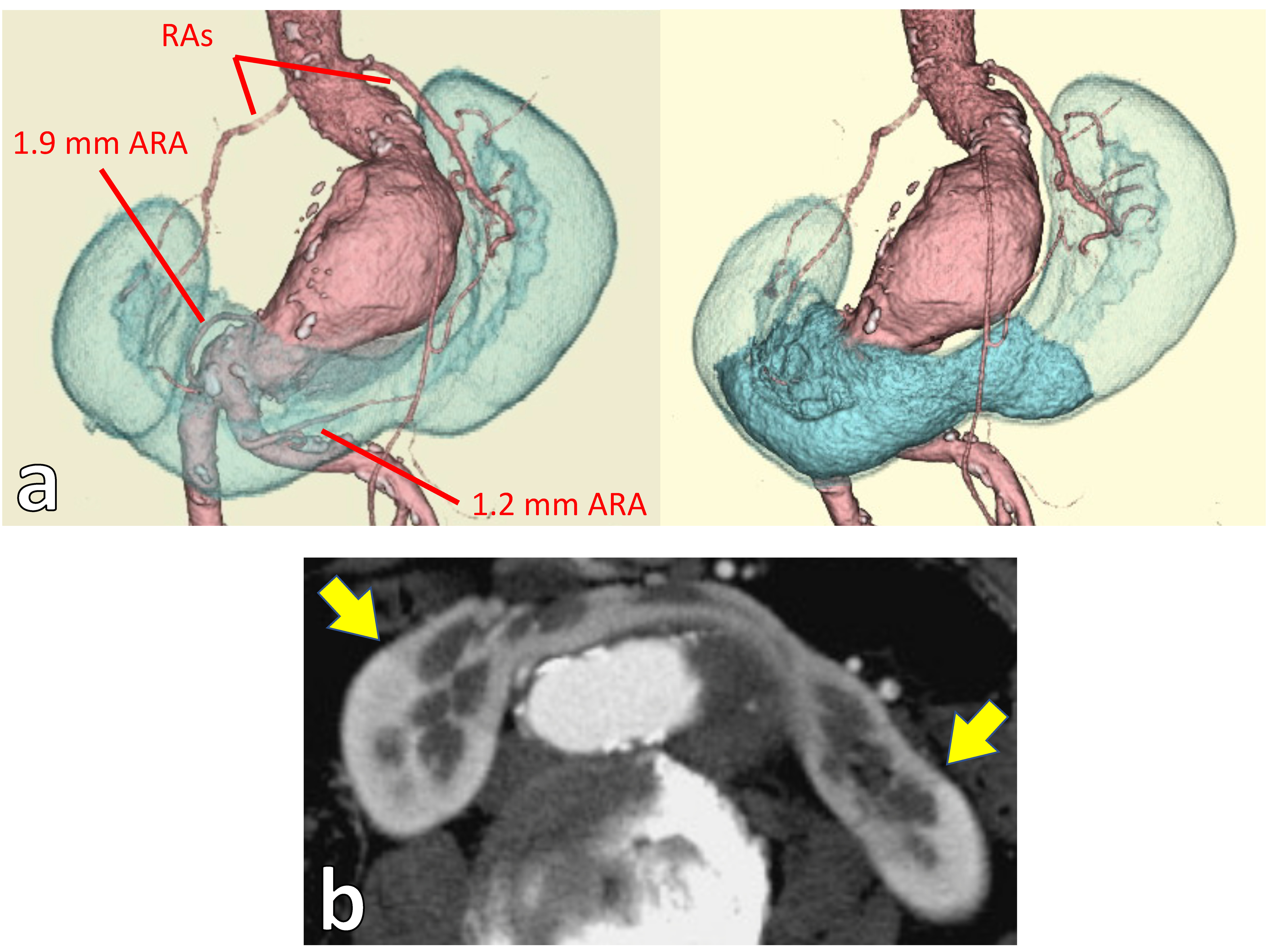
Fig. 1 Preoperative contrast-enhanced computed tomography and volumetry of the isthmus. (**a**) Preoperative three-dimensional reconstruction shows an infrarenal abdominal aortic aneurysm with accessory renal arteries originating from the aortic aneurysm and left common iliac artery. The deep blue area is the isthmus, which is between watershed lines, and it is supplied by accessory renal arteries. RAs: renal arteries; ARA: accessory renal artery. (**b**) Preoperative contrast-enhanced computed tomography shows clear watershed lines (arrows) at the margin of the isthmus. The calculated volume of the isthmus was 24.5% (94.15 cm^3^) of the total parenchyma (384.87 cm^3^).

**Figure 2:**
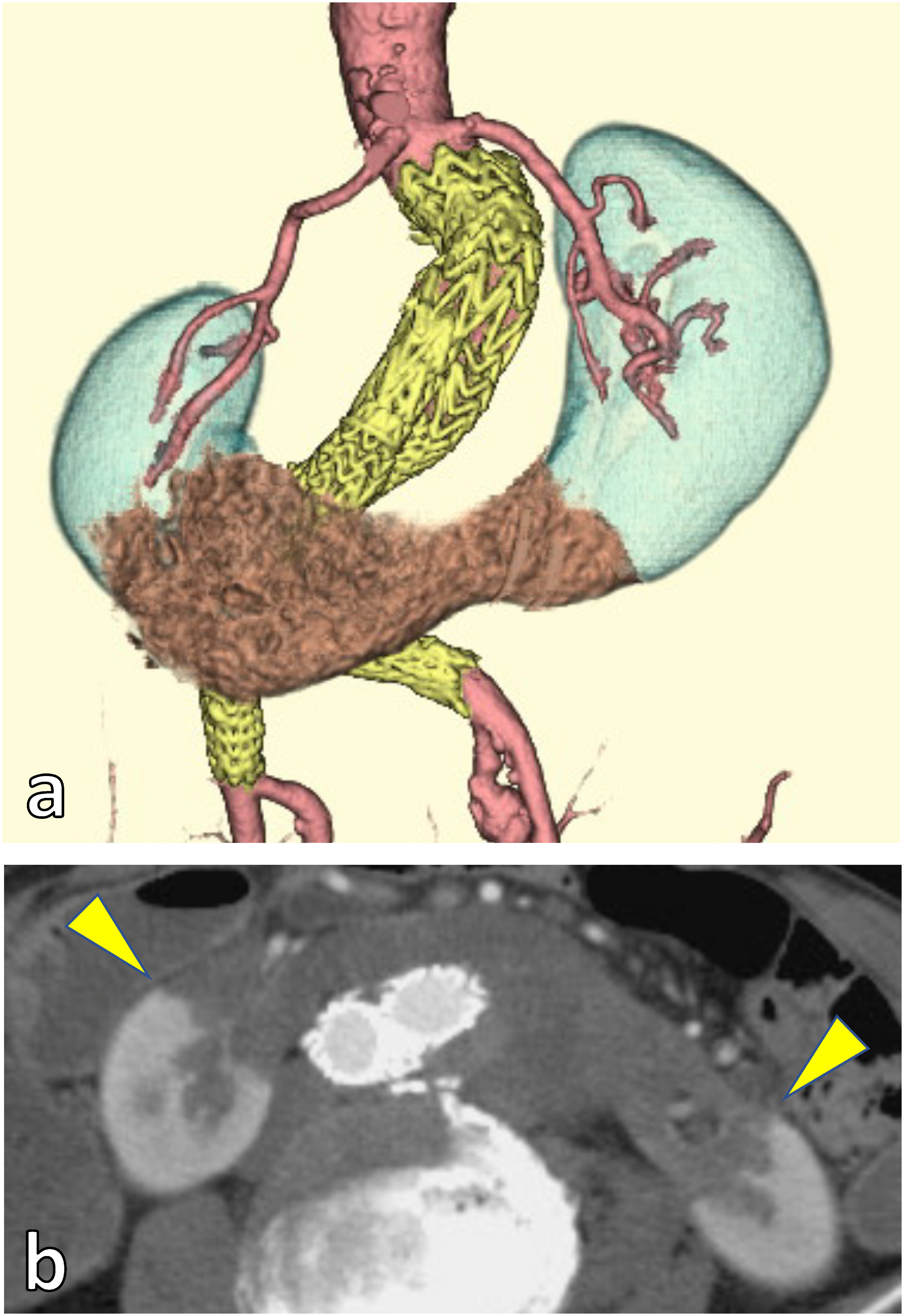
Fig. 2 Postoperative contrast-enhanced computed tomography and ischemia of the isthmus. (**a**) Postoperative three-dimensional reconstruction shows endovascular abdominal aortic aneurysm repair and ischemia of the isthmus (brown area) by occlusion of accessory renal arteries. (**b**) Postoperative contrast-enhanced computed tomography shows infarction of the isthmus in the same area between the watershed lines (arrow heads). The estimated infarction volume of the isthmus was 24.2% (84.18 cm^3^) of the total parenchyma (384.87 cm^3^).

A 75-year-old man with HSK was referred for treatment of a 59-mm infrarenal AAA. He had a medical history of hypertension, colon polyps, and chronic cholecystitis. Laboratory data showed that his renal function was normal (creatinine level: 0.65 mg/dL, estimated glomerular filtration rate: 87 mL/min/1.73 m^2^). Preoperative CECT showed an infrarenal AAA diameter of 59 mm and HSK. The HSK had two normally positioned main renal arteries on both sides of the kidney and two ARAs on the isthmus from the aortic aneurysm (1.9 mm in diameter) and the left common iliac artery (1.2 mm in diameter) (**Fig. 1a**). The patient’s anatomy was suitable for EVAR. Open repair of AAA in patients with HSK is associated with significant procedural risks because of the challenges posed by adequate exposure of the aneurysm and renal vasculature.^2)^ Because of additional considerations of the patient’s age and frailty, we decided that his AAA with HSK should be treated by EVAR. Preoperative CECT also showed clear watershed lines at the margin of the isthmus, which was perfused by the ARAs (**Fig. 1b**). ARAs that supply up to 32% of the total parenchyma can be occluded in patients with normal renal function.^3)^ In our patient, the calculated volume of the isthmus using CECT was 24.5% (94.15 cm^3^) of the total parenchyma (384.87 cm^3^) (**Fig. 1**). This finding indicated that occlusion of the ARAs by EVAR would not affect postoperative renal function. Because of accurate preoperative renal volumetry, there was no need to perform selective angiography of the ARAs to measure the volume of the isthmus. Endovascular exclusion of the aneurysm was performed by implanting a bifurcated Excluder endograft with an aortic extender and an iliac extension (W. L. Gore, Flagstaff, AZ, USA), which was deployed between the distal area of the main renal arteries and the end of both common iliac arteries. This procedure was associated with exclusion of the ARAs (**Fig. 2a**). After the operation, the patient suffered from mild lower abdominal pain that was induced by infarction of the isthmus, and this pain was relieved in 3 days. Postoperative renal function was not impaired (creatinine level: 0.74 mg/dL, estimated glomerular filtration rate: 78 mL/min/1.73 m^2^). Postoperative CECT showed infarction of the isthmus in the same area between the watershed lines without any type 2 endoleak (**Fig. 2b**). The volume of the renal infarction was almost equal (84.18 cm^3^) to that supplied by the ARAs preoperatively (**Fig. 2**). The estimated infarction volume of the isthmus was 24.2% of the total parenchyma (384.87 cm^3^). The patient’s postoperative course was uneventful, and he was discharged home 5 days after surgery.

## Discussion

HSK is an uncommon urological congenital anomaly of renal development. HSK occurs in approximately 0.25% of the population^4)^ and is thought to be present in only 0.12% of all cases of AAA.^1)^ The surgical open approach by graft replacement of AAA for patients with HSK requires different strategies for ligation or reconstruction of the ARAs and preservation or division of the isthmus.^2)^ EVAR is a safe method of treating AAA. A previous study using selective ARA angiography with spiral CT showed that ARAs supplying <32% of the total parenchyma could be occluded by an endovascular graft without postoperative renal dysfunction.^3)^ Attention should be paid to patients with preoperative renal failure. Fabiani et al. reported an 81-year-old patient with previous renal failure (creatinine level: 1.8 mg/dL) who required 3 weeks of hemodialysis after EVAR, which occluded two ARAs to the isthmus.^5)^ By using selective angiography of the ARAs with/without a CT scan, the patient’s postoperative renal function can be predicted by calculating the volume of the estimated area of renal isthmus infarction, which is caused by occlusion of the ARAs. However, this angiography is an invasive examination for patients, including the possibility of embolization and vascular injury. Additionally, Jackson et al. found that because of the low sensitivity of preoperative angiography for detecting ARAs, a satisfactory performance of selective ARA angiography may only be possible in less than half of the patients.^6)^ Preoperative CECT without selective angiography in the current case showed clear watershed lines between the areas supplied by the renal arteries and ARAs. This sign might have been caused by the two following possibilities. First, there was no vascular communication between the renal arteries and ARAs. Second, there were different blood flow velocities in the renal arteries, which were located above the abdominal aneurysm, and in the ARAs, which were located under the aneurysm. The watershed lines sign shown by preoperative CECT in a patient with AAA and HSK may enable the calculation of an accurate volume of renal isthmus infarction induced by occlusion of the ARAs and the estimation of a postoperative renal function, without any invasive examinations, such as selective renal artery angiography. In our case, the patient’s renal function was normal (estimated glomerular filtration rate: 87 mL/min/1.73 m^2^), and the estimated renal infarction volume was 24.2% of the total parenchyma. EVAR was safely performed in our patient, and postoperative renal dysfunction did not occur. The estimated postoperative residual renal volume was 290.72 cm^3^ (75.5%), and the predicted postoperative estimated glomerular filtration rate was 66 mL/min/1.73 m^2^. The actual postoperative residual renal volume was 300.69 cm^3^ (75.8%), which was as expected, but the actual postoperative estimated glomerular filtration rate was 78 mL/min/1.73 m^2^, which was higher than expected. Intraoperative angiography showed no findings suggestive of abnormal blood flow in the ARAs, but the isthmus might have had less blood flow than other parts of the kidney. Additionally, the isthmus may contain connective tissue and renal parenchyma.^7)^ Therefore, a simple proportional calculation of renal function and renal volume might not be appropriate. Further studies are required in patients with AAA and HSK with preoperative CECT to determine the exact mechanism of this watershed sign. In addition, we should pay more attention to preoperative CECT scans in patients with AAA and HSK. In the future, we hope to analyze multiple imaging findings of the HSK and provide data on the watershed line and the volumetry, as well as determine the degree of universality of renal function.

## Conclusion

We report a case of EVAR for a patient with HSK, and preoperative CECT showed the interesting sign of watershed lines, which enabled the calculation of the isthmus volume. On the basis of this volumetry and the patient’s preoperative renal function, EVAR was safely performed without impairment of postoperative renal function.

## Data Availability

The authors declare that all data in this article are available within the article.

## Abbreviations

HSK: horseshoe kidney
AAA: abdominal aortic aneurysm
EVAR: endovascular aneurysm repair
ARA: accessory renal artery
CE: contrast-enhanced
CT: computed tomography
